# A real-world study of the efficacy of second-line treatment of unresectable hepatocellular carcinoma with esophagogastric varices after progression on first-line lenvatinib combined with PD-1 inhibitor

**DOI:** 10.1186/s12957-025-03742-0

**Published:** 2025-03-13

**Authors:** Saifeng Li, Qin Wen, Wenwu Huang, Zeyu Qiu, Long Feng, Fengming Yi

**Affiliations:** 1 Department of Oncology, Second Affiliated Hospital of Nanchang University, Minde Road 1, Nanchang, 330006 P. R. China; 2 Jiangxi Medical College of Nanchang University, Nanchang, 330006 P. R. China

**Keywords:** Hepatocellular carcinoma, Second line, Lenvatinib, PD-1 inhibitor

## Abstract

**Purpose:**

The incidence and mortality of hepatocellular carcinoma are still high according to National Cancer Center of China. Atezolizumab plus bevacizumab has become one of the standard regimens for the first-line treatment of unresectable hepatocellular carcinoma. However, some patients still use lenvatinib in combination with immunotherapy instead of a standard “atezolizumab-bevacizumab” regimen as a lower risk of bleeding in patients with esophagogastric varices. However, there is no evidence for second-line therapy after progression on lenvatinib combined with PD-1 inhibitor in unresectable hepatocellular carcinoma till now. Herein, we aim to investigate second-line treatment among these patients.

**Patients and methods:**

Thirty-three patients with unresectable hepatocellular carcinoma with esophagogastric varices were admitted to the Second Affiliated Hospital of Nanchang University from January 2019 to December 2023. They were treated with lenvatinib in combination with PD-1 inhibitor as first line. The efficacy was conducted according to the RECIST1.1 criteria. The endpoints included objective response rate (ORR), disease control rate (DCR), median overall survival (OS), and median progression free survival (PFS).

**Results:**

We identified a total of 225 patients with unresectable hepatocellular carcinoma with esophagogastric varices who received first-line lenvatinib in combination with PD-1 inhibitor, of whom 33 (14.7%) received second-line therapy. 21 patients (63.6%) were treated with regorafenib combined with PD-1 inhibitor, 6 patients (18.2%) with apatinib plus PD-1 inhibitor, 4 patients (12.1%) with bevacizumab plus PD-1 inhibitor, and the remaining 2 patients with regorafenib or sorafenib as monotherapy, respectively. Of the 33 patients, 2 (6.1%) were evaluated as partial response (PR), 16 (48.5%) had stable disease (SD), and 15 (45.4%) experienced progression (PD). The ORR was 6.1%, and the DCR was 54.6%. Median PFS was 4.5 months, median OS was 7.2 months, and the 12-month OS rate was 27.3%. Overall survival follow-up was done in 37 patients without second line treatment whose baseline levels were matched with those of the treatment group. The OS was 7.2 months in second line treatment group versus 3.0 months in control group (*p* = 0.04). As for different treatments in a second line, The ORR of regorafenib in combination with PD-1 inhibitor was 9.5%, the DCR was 47.6%, the median PFS was 4.2 months, and the median OS was 5.9 months. None of the patients treated with apatinib plus PD-1 inhibitor got PR, the DCR was 83.3%, the median PFS was 8.7 months, and the median OS was 9.1 months. None of the patients treated with bevacizumab plus PD-1 inhibitor got PR, the DCR was 25.0%, the median PFS was 2.2 months, and the median OS was 6.0 months.

**Conclusion:**

The second-line treatment of unresectable hepatocellular carcinoma with esophagogastric varices after progression on first-line lenvatinib combined with PD-1 inhibitor is effective. Regorafenib or apatinib combined with PD-1 inhibitor might be the preferred options.

## Introduction

Hepatocellular carcinoma (HCC) is one of the common malignant tumors, ranking the sixth in the morbidity and the third leading cause of death among malignant tumors in the world [1]. The onset of HCC is relatively insidious, and nearly 70% of patients are diagnosed in the intermediate or advanced stage without the opportunity of radical resection. Currently, the first-line systemic treatment includes atezolizumab-bevacizumab(A/B), durvalumab-tremelimumab(D/T), sorafenib, lenvatinib, or durvalumab [2]. However, patients with esophageal or gastric varices were excluded from atezolizumab-bevacizumab [3]. lenvatinib plus pembrolizumab was identified as another option of first line, with high objective response rate according to keynote-524 [4], although a randomized, double-blind, phase 3 trial indicated negative results [5]. Numerous studies proved that lenvatinib combined with PD-1 inhibitor was an alternative strategy for patients with advanced HCC [6–9]. Moreover, lenvatinib plus PD-1 inhibitor combined with locoregional treatment including hepatic arterial infusion chemotherapy (HAIC), transcatheter arterial chemoembolization (TACE), radiotherapy, or microwave ablation (MWA) were evaluated as effective treatment for patients with advanced HCC [10–13].

The second-line treatment for advanced HCC is controversial in the current preferred immune-oncology based strategy. Regorafenib, cabozantinib, or ramucirumab are recommended as standard second line treatments after sorafenib progression [2, 14–16]. Lenvatinib has been validated to be effective second line option after A/B first line progression with numerous retrospective studies [17–20]. Regorafenib monotherapy or regorafenib plus PD-1 inhibitor was also identified as alternative second line option after A/B first line progression although without solid evidence [21, 22]. For HCC patients with esophagogastric varices, A/B was not suitable as the preferred option as the risk of bleeding, then lenvatinib plus PD-1 inhibitor might be one of the alternative solutions. However, second line treatment after lenvatinib plus PD-1 inhibitor progression in HCC patients with esophagogastric varices has not been reported till now. Herein, we aim to investigate the efficacy of the second line treatment after lenvatinib plus PD-1 inhibitor progression for the patients with unresectable HCC complicated with esophagogastric varices.

## Materials and methods

Patients with unresectable hepatocellular carcinoma with esophagogastric varices were collected at the Second Affiliated Hospital of Nanchang University from January 2019 to December 2023, and a total of 33 patients were included in this study. The inclusion criteria for this study were as follows: aged was between 18 and 75 years old; pathologically confirmed hepatocellular carcinoma or met the clinical diagnostic criteria HCC [23]; Advanced stage of liver cancer that was not eligible for surgical resection; previous first line treatment was lenvatinib plus PD-1 inhibitor and tumor progressed; Eastern Cooperative Oncology Group (ECOG) was lower than 2; Child-Pugh liver function was ≤ 7. Patients with a previous or concurrent history of other malignant tumors or patients treated with second line after lenvatinib plus PD-1 inhibitor returned to hospital without evaluation as disease progression or physical status decreased were excluded. Patients were treated with second-line regimen. Radiological examination was routinely performed every 6 weeks. Tumor response was evaluated according to RECIST1.1. Complete response (CR) refers to the complete disappearance of all target lesions. Partial response (PR) indicates the sum of the diameters of all target lesions should be reduced by at least 30% of total diameter at baseline. Progressive disease (PD) is the total diameter of all target lesions has increased by at least 20% of total diameter at baseline. Stable disease (SD) indicates the total diameter of all target lesions is between PR and PD. Objective response rate (ORR) equals to CR + PR. Disease control rate (DCR) is CR + PR + SD. Adverse reactions were assessed according to Common Terminology Criteria for Adverse Events 4.0 (CTCAE4.0).

### Statistical analysis

This is a retrospective study without any intervention. Data collected was based on real world investigation. Data were analyzed using SPSS 26.0 and GraphPadPrism 9. The waterfall plot of response from baseline was drawn by SPSS26.0. Median progression free survival (PFS) and median overall survival (OS) were analyzed by Kaplan-Meier.

## Results

### Baseline characteristics of patients recruited

We identified a total of 225 patients with unresectable hepatocellular carcinoma with esophagogastric varices who received first-line lenvatinib in combination with PD-1 inhibitor, of whom 33 (14.7%) received second-line therapy, including 30(90.9%) males and 3(9.1%) females. Obviously, the proportion of second line treatment after lenvatinib plus PD-1 inhibitors was low in our center, and the reason might be rapid progression or low cost-effectiveness of second line treatment in our not rich region. The median age was 52.0 years old; 16(48.5%), 29(87.9%), and 15(45.5%) patients were treated with surgery, TACE, and HAIC before. All the patients were Child-pugh A; median alpha fetoprotein (AFP) level was 489.8ng/ml; 14(42.4%) patients were complicated with macrovascular invasion; and distant metastasis emerged in 27(81.8%) patients; 21(63.6%) patients were hepatitis B infection. 21 patients (63.6%)were treated with regorafenib combined with PD-1 inhibitor as the combination of regorafenib and PD-1 inhibitors showed a synergistic effect and superiority than regorafenib monotherapy [24]. 6 patients (18.2%) with apatinib plus PD-1 inhibitors as camrelizumab plus apatinib showed promising efficacy in first or second line setting in advanced HCC [25, 26]. 4 patients (12.1%) with bevacizumab plus PD-1 inhibitors as bevacizumab plus PD-1 inhibitors demonstrated high response rate and better overall survival in first line setting in unresectable HCC [27, 3]. and the remaining 2 patients with regorafenib or sorafenib as monotherapy. The details of baseline features of patients enrolled were presented in Table 1.

**Table 1:** Baseline characteristics of patients enrolled

	Total(*n* = 33)
Median age	52.0(45.0,61.5)
Sex, n (%)	
Male	30 (90.9)
Female	3 (9.1)
Previous treatment, n (%)	
Surgery	16 (48.5)
TACE	29 (87.9)
HAIC	15 (45.5)
Child-pugh A, n (%)	33(100)
AFP	489.8 (28.3,3451.0)
Macrovascular invasion (MVI), n (%)	
Absent	19 (57.6)
Present	14 (42.4)
Distant metastasis, n (%)	
Absent	6 (18.2)
Present	27 (81.8)
Hepatitis B infection, n (%)	21 (63.6)
Second line treatment, n (%)	
Regorafenib + PD-1 inhibitor	21 (63.6)
Apatinib + PD-1 inhibitor	6 (18.1)
Bevacizumab + PD-1 inhibitor	4 (12.1)
Regorafenib monotherapy	2(6.1)
Sorafenib monotherapy	2(6.1)

Abbreviations: TACE, transcatheter arterial chemoembolization; HAIC, hepatic arterial infusion chemotherapy; AFP, alpha-fetoprotein

### Efficacy

**Fig. 1:**
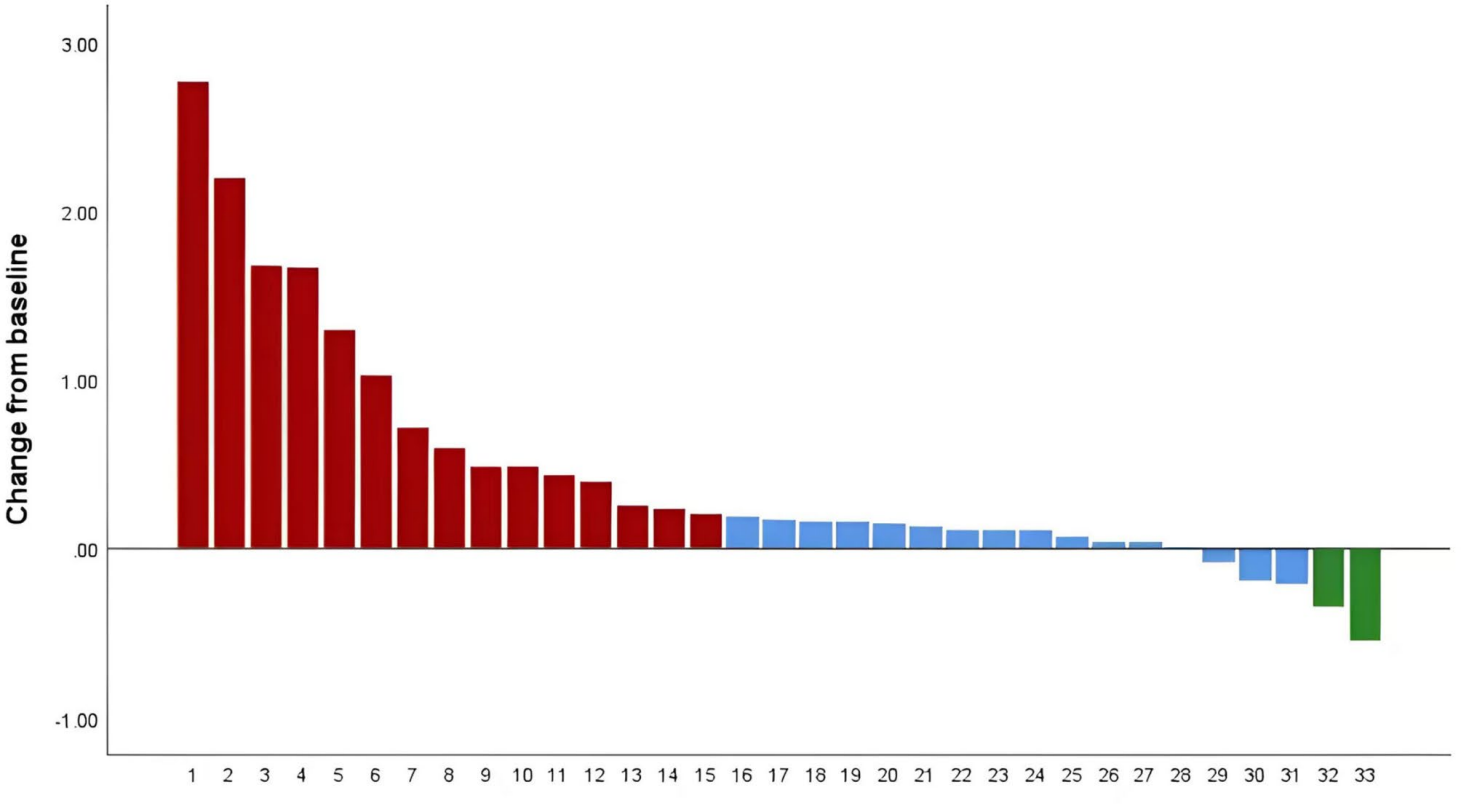
Efficacy of HCC patients treated with second line after Lenvatinib and PD-1 inhibitor progression. Red indicates PD; Blue indicates SD, Green indicates PR

**Fig. 2:**
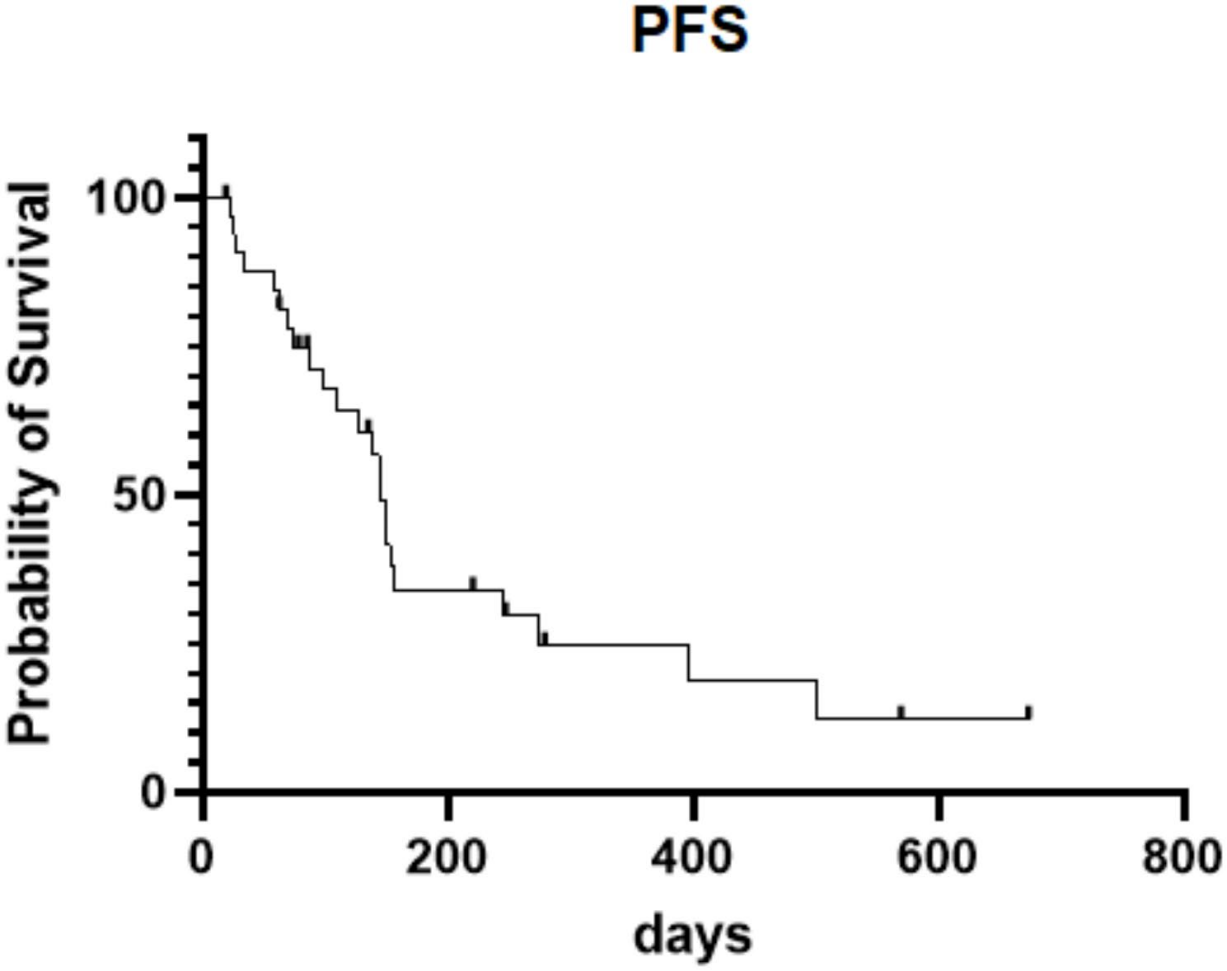
Progression-free survival of patients treated with second line regimen after progression of lenvatinib and PD-1 inhibitor as first line treatment

**Fig. 3:**
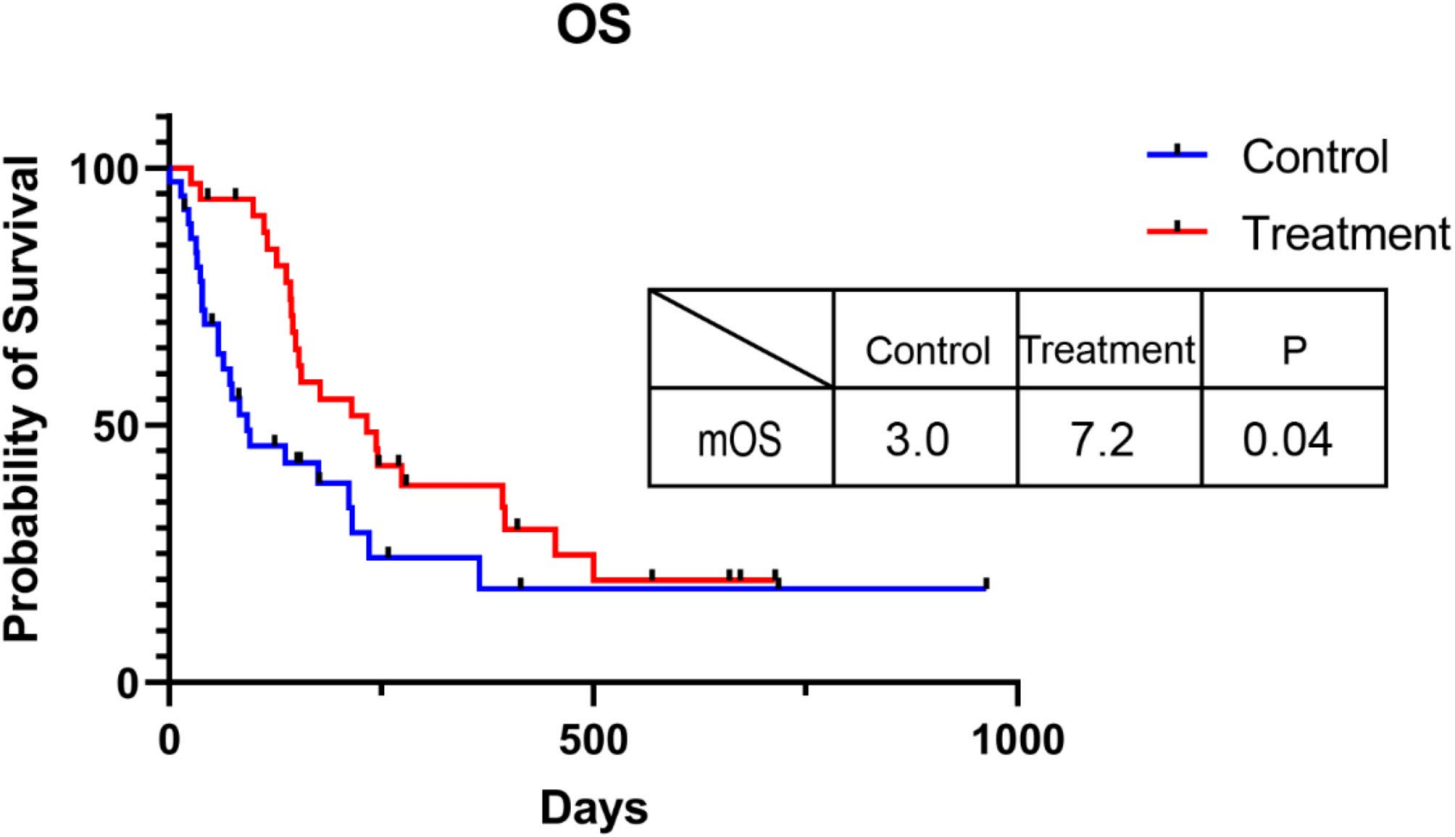
Overall survival comparison of patients treated with second line regimen or not after progression of lenvatinib and PD-1 inhibitor as first line treatment

Of the 33 patients, 2 (6.1%) were evaluated as PR, 16 (48.5%) had SD, and 15 (45.4%) experienced PD. The ORR was 6.1%, and the DCR was 54.6%. Median PFS was 4.5 months, median OS was 7.2 months, and the 12-month OS rate was 27.3%. The details were presented in Table 2; Figs. 1, 2 and 3. Overall survival follow-up was done in 37 patients without second line treatment whose baseline levels were matched with those of the treatment group. The OS was 7.2 months in second line treatment group versus 3.0 months in control group (*p* = 0.04) (Fig. 3). Obviously, the efficacy of second line treatment after lenvatinib plus PD-1 inhibitors is limited, but it significantly improved overall survival when compared to the non-treatment group.

**Table 2:** The efficacy of the second line treatment after lenvatinib plus PD-1 inhibitor progression for patients enrolled

Evaluation	*n*	%
Overall tumor evaluation		
CR PR SD PD ORR DCR	0 2 16 15 2 18	0 6.1 48.5 45.4 6.1 54.6

Abbreviations: CR, complete response; PR, partial response; SD, stable disease; PD, progressive disease; DCR, disease control rate; ORR, objective response rate

As for different treatments, The ORR of regorafenib in combination with PD-1 inhibitor was 9.5%, the DCR was 47.6%, the median PFS was 4.2 months, and the median OS was 5.9 months. The combination of regorafenib plus PD-1 inhibitor was comparable with regorafenib monotherapy in RESORCE study [14]. The crossline treatment of PD-1 inhibitor didn’t show any superiority in these patients, although the number of patients recruited was limited. None of the patients treated with apatinib plus PD-1 inhibitor got PR, the DCR was 83.3%, the median PFS was 8.7 months, and the median OS was 9.1 months. The combination of apatinib plus PD-1 inhibitor indicated high DCR in current study although the median PFS or OS was limited, and the result was comparable with apatinib monotherapy as second line treatment [15]. It might be another option for these patients. None of the patients treated with bevacizumab plus PD-1 inhibitor got PR, the DCR was 25%, the median PFS was 2.2 months, and the median OS was 6.0 months.

### Safety

Adverse reactions were evaluated according to CTCAE4.0. Among the 33 patients, four patients developed grade 3 or above urinary protein, four patients suffered digestive bleeding, and one patient experienced PD-1 related cutaneous reaction or hepatitis, respectively. All the adverse effects were under control, and no treatment-related death was observed in the second line treatment.

## Discussion

Currently, atezolizumab plus bevacizumab or tremelimumab plus durvalumab was recommended as preferred options for first line treatment of advanced hepatocellular carcinoma [28, 29], while patients with esophagogastric varices were excluded in atezolizumab plus bevacizumab group. Lenvatinib monotherapy was one of alternative options for unresectable HCC [30]. However, Lenvatinib plus pembrolizumab didn’t significantly improve OS and PFS versus lenvatinib plus placebo, although earlier study Keynote-524 showed promising antitumor activity in the first line treatment with an ORR of 36.0%^4,5^. Lenvatinib plus anti-PD-1 antibodies indicated promising efficacy and tolerable regimen as conversion treatment in unresectable HCC [9]. The combination of transarterial chemoembolization and lenvatinib plus anti-PD-1 antibodies was also demonstrated a high response rate and recommended as an option for conversion therapy in initially unresectable hepatocellular carcinoma [31]. Moreover, hepatic arterial infusion chemotherapy (HAIC) or TACE combined with PD-1 inhibitors plus lenvatinib showed promising results in patients with advanced HCC [10, 31–34]. Therefore, PD-1 inhibitors plus Lenvatinib was an alternative option for patients with HCC, especially complicated with esophageal-gastric fundus varices.

A multicenter, retrospective study investigated second line treatment after A/B as first line in patients with advanced HCC and demonstrated that continuation of active therapy after A/B progression indicated better survival [35]. Another two studies also confirmed that second line treatment after Lenvatinib monotherapy progression was effective [36, 37]. Second-line hepatic arterial infusion chemotherapy after atezolizumab-bevacizumab failure in HCC showed survival improvement [38]. However, second line therapy after lenvatinib plus PD-1 inhibitors progression in unresectable HCC was unknown till now. The effectiveness of the second line treatment after Lenvatinib plus PD-1 inhibitor was confirmed in our study as well, and the OS was 7.2 months in second line treatment group versus 3 months in non-treatment group after the failure of Lenvatinib plus PD-1 inhibitor. Therefore, second line treatment could improve survival in different first line treatment options.

As for second line treatment options in the era of immunotherapy, there was no consensus or standard till now. Regorafenib, cabozantinib, or ramucirumab were recommended as standard second line treatments after sorafenib progression [2, 14–16]. Second line treatment with multikinase inhibitors including sorafenib, lenvatinib, and cabozantinib in patients with advanced HCC after disease progression on atezolizumab-bevacizumab was effective, although the ORR and DCR were 6.1% and 63.3%, respectively [39]. Ramucirumab demonstrated a 10.6% ORR, and 8.7 months OS in second line treatment for patients with HCC following Non-Sorafenib Systemic Therapy [40]. Our research was in line with the above-mentioned studies. The ORR was 6.1%, and the DCR was 54.6%. Median PFS was 4.5 months, median OS was 7.2 months, and the 12-month OS rate was 27.3% in current study. Therefore, the effectiveness of second line treatment could reach 6.1-10.6% ORR and nearly 54.6-63.3% DCR, and the OS was more or less 8 month in immunotherapy as first line therapy. Previous studies indicated that regorafenib and PD-1 inhibitors showed a synergistic effect and superiority than regorafenib monotherapy [24]. apatinib plus PD-1 inhibitors as camrelizumab plus apatinib showed promising efficacy in first or second line setting in advanced HCC [25, 26]. Then we chose regorafenib plus PD-1 inhibitor, apatinib plus PD-1 inhibitor, bevacizumab plus PD-1 inhibitor, regorafenib or sorafenib monotherapy as the second line treatment options after Lenvatinib plus PD-1 inhibitor progression. The final result showed that the ORR of regorafenib in combination with PD-1 inhibitor was 9.5%, the DCR was 47.6%, the median PFS was 4.2 months, and the median OS was 5.9 months. None of the patients treated with apatinib plus PD-1 inhibitor got PR, the DCR was 83.3%, the median PFS was 8.7 months, and the median OS was 9.1 months. None of the patients treated with bevacizumab plus PD-1 inhibitor got PR, the DCR was 25%, the median PFS was 2.2 months, and the median OS was 6.0 months. The combination of regorafenib plus PD-1 inhibitor was comparable with regorafenib monotherapy in RESORCE study [14]. The crossline treatment of PD-1 inhibitor didn’t show any superiority in these patients, although the number of patients recruited is limited. The combination of apatinib plus PD-1 inhibitor indicated high DCR in current study although the median PFS or OS was limited, and the result was comparable with apatinib monotherapy as second line treatment [15]. It might be another option for these patients.

Taken together, the second line treatment after progression on first-line lenvatinib combined with PD-1 inhibitor was effective, but overall effect was limited. Regorafenib combined with PD-1 inhibitor might be the preferred regimen, and apatinib combined with PD-1 inhibitor was an alternative option for second line treatment after Lenvatinib plus PD-1 inhibitor progression. Randomized controlled studies or studies enrolled large numbers of patients should be carried out to verify this conclusion. As for safety, in our study, most of the patients were tolerable without severe adverse effects. However, the current study has limitations. Firstly, this study was retrospective, and there may be some confounding factors that may affect the treatment efficacy; secondly, only 33 cases included in final analysis, and further studies with larger sample size were needed in the future.

## Data Availability

No datasets were generated or analysed during the current study.
